# Effect of Home‐Based Multiple Micronutrient Powder Fortification on Haemoglobin Levels in Infants in Nampula, Mozambique: A Pragmatic Clinical Trial

**DOI:** 10.1111/mcn.70210

**Published:** 2026-06-19

**Authors:** Ana Raquel Ernesto Manuel Gotine, Marly Augusto Cardoso

**Affiliations:** ^1^ Postgraduate Program in Public Health University of São Paulo (USP) São Paulo São Paulo Brazil; ^2^ Department of Nutrition, Faculty of Health Sciences Lurio University Nampula Mozambique; ^3^ Department of Nutrition School of Public Health São Paulo São Paulo Brazil; ^4^ Global Health and Tropical Medicine, GHTM, Associate Laboratory in Translation and Innovation Towards Global Health, LA‐REAL, Instituto de Higiene e Medicina Tropical, IHMT, Universidade NOVA de Lisboa, UNL Lisboa Portugal

**Keywords:** anaemia, complementary feeding, home fortification, iron deficiency, micronutrients

## Abstract

To conduct a pragmatic clinical trial with multiple micronutrient powders (MNPs) for anaemia prevention during the first year of life in primary care in Nampula, Mozambique. A total of 275 infants, aged 6–8 months, were randomly assigned to intervention (IG, *n* = 145) and control (CG, *n* = 130) groups. The IG received 90 sachets of MNP for daily use up to 180 days; the CG received standard care. Haemoglobin (Hb) was measured at baseline and after 6 months. Analysis used linear regression adjusted for child and maternal variables (*p* < 0.05). At baseline, mean Hb (95% CI) was 102.2 g/L (100.4–104.0) in the IG and 103.8 g/L (102.0–105.7) in the CG, with corresponding anaemia rates (Hb < 105 g/L) of 55.2% (47.1; 63.0%) and 47.7% (39.3; 56.2%). After intervention, IG had 106.2 g/L (104.2–108.1) vs CG 99.5 g/L (97.3–101.8), with anaemia rates of 36% (27.8; 45.1) and 67.3% (57.9; 75.4), respectively. Adjusted mean intra‐group difference of haemoglobin (g/L) from baseline to follow‐up was +2.8 in IG and −4.3 in CG. MNPs improved haemoglobin levels, supporting their potential for anaemia control in vulnerable populations.

**Trial Registration:**

https://ensaiosclinicos.gov.br/rg/RBR-4r37q7z. Registered 11/11/2024.

## Summary

- Home fortification with multiple micronutrient powders (MNP) has an impact on improving haemoglobin levels and reducing anaemia in infants.
- Adequate adherence to the use of MNPs enhances their beneficial effects.
- The high prevalence of childhood anaemia in Nampula reinforces the urgency of combined prevention strategies.
- This is the first pragmatic clinical trial with MNPs conducted in Mozambique.

## 1 Introduction

Anaemia is a serious global public health problem in children 6–59 months of age (World Health Organization 2011). The World Health Organization (WHO) has recently updated the haemoglobin cutoff values for anaemia: < 105 g/L in children 6–23 months, and < 110 g/L for 24–59 months (World Health Organization 2024). Iron deficiency is one of the main factors contributing to the global anaemia burden, predominantly affecting vulnerable populations in low‐ to‐middle income countries (Pasricha et al. 2021). The condition affects an estimated 40% of children under 5 years and 37% of pregnant mothers worldwide (Irineu et al. (2023), with the highest prevalence in Africa, where over 60% of infants and around 50% öf pregnant mothers are affected (UNICEF 2023). These high rates illustrate the major challenge in meeting the Sustainable Development Goal (SDG) 2.2, which aims to end malnutrition by 2030 and improve nutrition in vulnerable groups by 2025 (United Nations 2015).

In Mozambique, anaemia affects 73% of children aged 6–59 months (Ministério da Saúde de Moçambique MISAU 2023), where Nampula, an endemic area for malaria, numbers among one of the worst affected provinces (Mozambique Ministry of Health 2019). Anaemia rates decline steadily with age, with the condition mainly affecting children aged 6–24 months in poorer families. The leading cause of deficiency and anaemia is low intake of micronutrients such as iron, vitamin A, folate, and vitamin B1 (Ministério da Saúde de Moçambique MISAU 2023) (Mozambique Ministry of Health 2019). Malaria constitutes the most common complication associated with anaemia (Instituto Nacional de Saúde 2019). Infection by Plasmodium leads to rapid destruction of red blood cells both directly, via rupture of infected cells (intravascular haemolysis), and indirectly, by altering healthy red blood cells, which are then removed by the spleen (extravascular haemolysis) (White 2018). Moreover, the inflammatory response to infections inhibits the production of fresh red blood cells by bone marrow, further contributing to the decline in haemoglobin levels(Gaston et al. 2021) (Dao et al. 2021).

Home fortification of foods with multiple micronutrient powders (MNPs) is recommended by the WHO as an effective public health strategy to reduce anaemia and iron deficiency in children under 2 years of age (Belachew and Tewabe 2020; Oliveira et al. 2016). Evidence from systematic reviews shows that MNP use reduces anaemia by approximately 30%–34% and iron deficiency by up to 51%–57% among infants and young children in low‐income settings (De‐Regil et al. 2011; De‐Regil et al. 2017; Salam et al. 2013). Effectiveness studies conducted within routine health services further support these findings. For example, in Bihar, India, home fortification within a government programme led to modest improvements in haemoglobin concentrations and reductions in anaemia and diarrhoea prevalence (Young et al. 2021). Similar benefits have been observed among young Brazilian children receiving MNPs through primary healthcare services (Cardoso et al. 2016).

The World Health Organization first issued a guideline in 2011 recommending home fortification of complementary foods with MNPs containing iron, vitamin A, and zinc for children aged 6–23 months (World Health Organization 2011). This recommendation was further reinforced in 2017, when updated guidance emphasized point‐of‐use fortification as a strategy to prevent and treat anaemia and iron deficiency in settings where anaemia prevalence among young children is 20% or higher and constitutes a major public health concern (World Health Organization 2017). The high prevalence of anaemia and childhood malnutrition in Mozambique, combined with health system and contextual challenges that may influence the implementation of nutritional interventions, underscores the need to assess the impact of home fortification with MNPs within routine primary healthcare services. Although pragmatic trials of MNPs have been conducted in other settings, there is limited evidence on their real‐world impact in the Mozambican context. To the best of our knowledge, this is the first pragmatic clinical trial conducted in Mozambique to evaluate the impact of MNPs on haemoglobin levels in infants, aiming to generate locally relevant evidence to inform strategies for anaemia prevention in primary care.

## 2 Methods

### 2.1 Study Design and Venue

The study was conducted in Nampula, a province located in the northern part of Mozambique, situated around 2150 km north of the country's capital city, Maputo(Araújo 2012). Nampula province, divided into 23 districts, has an area of 81,606 Km² and a population of 6,102,867. Nampula city, the capital of Nampula province, is in the interior of the province and covers an area of 482 km^2^. According to the 2017 census, the city has a population of 663,212, comprising 325,373 men and 337,839 women (Araújo 2012; INE 2019).

The present study entailed a pragmatic clinical trial conducted at two health centres (*Saúde 25 de Setembro* and *Muhala Expansão*) in Nampula city, chosen for their greater coverage of more densely populated neighbourhoods with higher social vulnerability. Given the operational constraints of implementing the trial within routine primary health care services, the intervention was allocated at the health centre (cluster) level. A random draw was performed using the statistical program R version 3.6.1 to designate which health centre would receive routine dispensing of the sachets (intervention health centre) and which would receive standard care as provided under Mozambique´s National Health System (control health centre). Only two clusters were included in the study (one per study arm), which represents an important methodological limitation. In this design, the intervention effect is fully confounded with site‐specific characteristics, limiting the ability to disentangle intervention effects from contextual differences between health centres. Therefore, this study should be interpreted as providing preliminary, practice‐based evidence under real‐world conditions, rather than a definitive causal evaluation of effectiveness.

Data collection was carried out by a previously trained team comprising health professionals from the participating health centres (nurses, nutritionists, and nursing technicians) and final‐year students on Nutrition and Nursing programs of the University of Lurio. Recruitment was carried out among families who regularly attended health centres for routine child follow‐up consultations (growth and development consultations), in accordance with the regular childcare schedule adopted by the Ministry of Health. Thus, the participants were children seen in the usual context of primary health care. Families who attended the centres for regular child consultations, who lived within the geographical coverage radius of each health centre, and who met the inclusion criteria defined in the protocol were eligible. Intervention group participants each received 90 MNP sachets, dispensed in two stages, for use over a 180‐day period, as recommended by the WHO. Control group participants received advice on healthy complementary feeding and anaemia prevention as part of standard care by health professionals, with no dispensing of MNPs.

### 2.2 Inclusion and Exclusion Criteria

The caregivers of children 6–8 months of age, seen by the participating health centres, were invited to take part, provided the child was not taking iron supplements at time of recruitment. Exclusion criteria were children with a confirmed diagnosis of malaria by the rapid test for malaria (*First Response*® *Malaria Antigen P. falciparum (HRP2) Card Test)*, those with HIV infection, tuberculosis, hemoglobinopathies, pre‐term birth (< 37 weeks), presence of severe congenital disease, physical or mental disability. Children with severe anemia (haemoglobin < 70 g/L) or receiving treatment for anaemia at the time of recruitment were excluded and referred for management according to national guidelines.

### 2.3 Main Outcome and Sample Size Calculation

The sample size was estimated to detect a minimum clinically meaningful difference of 6 g/L in mean haemoglobin concentration between the intervention and control groups at follow‐up. Assuming a standard deviation of 12 g/L, a statistical power of 95%, and a two‐sided significance level of 5%, 105 children per group were required. To account for potential losses to follow‐up, 30% was added, resulting in a final target sample size of 135 children per group (Grimes and Schulz 2002). Participant recruitment was conducted sequentially among eligible children attending the health centres until the required sample size for each group was reached. Anaemia was defined using two haemoglobin cut‐off points: the traditional WHO threshold of < 110 g/L (World Health Organization 2011) and the updated threshold of < 105 g/L for children aged 6–23 months (World Health Organization 2024). Both definitions were applied in the analyses.

### 2.4 Data Collection and Procedures

At baseline, the research team conducted interviews with parents and/or caregivers, collecting data on socioeconomic and demographic conditions of the household, maternal characteristics, and child health profile and morbidities. Child weight and length were measured according to WHO recommended procedures(World Health Organization 2008). Nutritional status was classified according to the WHO guidelines, using the indices weight/age, weight/length and length/age expressed as Z‐scores(25. Weight measurements were obtained using a paediatric electronic balance (SECA 834), with a capacity of 16 kg and graduations of 10 g. Length was measured using a SECA portable infantometer, with a range of 10–100 cm and accuracy of 1 millimetre (0.1 cm). Dietary intake was assessed using a 24‐h dietary recall, in which mothers or caregivers reported all foods and beverages consumed by the child during the 24 h preceding the interview.

Haemoglobin concentrations were assessed at two time points (baseline and post‐intervention) by two trained health professionals who underwent prior standardization training according to Ministry of Health procedures. Capillary blood samples were obtained via finger prick using an aseptic technique. The first drop of blood was wiped away, and a subsequent free‐flowing drop was collected directly into a disposable microcuvette (approximately 10 µL) by capillary action. Samples were analysed immediately using a portable HemoCue Hb 201 DM haemoglobinometer. The device provides quantitative determination of haemoglobin in undiluted whole blood through photometric measurement at dual wavelengths (570 and 880 nm), with automatic turbidity compensation. The same standardized protocol was applied at both health centres to ensure consistency between operators. HemoCue is a rapid, well‐established and safe technique for assessing blood haemoglobin levels (Neufeld et al. 2002) (Anastasio et al. 2021). Rapid diagnostic tests for malaria were performed using the First Response Malaria Antigen P. falciparum (HRP2) Card Test.

A similar initial questionnaire was applied to both groups (control and intervention) at two timepoints—baseline (before group random draw) and post‐intervention— collecting socioeconomic information (household wealth index, calculated based on ownership of the following assets: radio, television, land‐line, computer/laptop/tablet, internet, electric iron, wood/coal‐burning stove, electric/gas stove, fridge/freezer, car, motorcycle, and bicycle (INE 2019; Hjelm et al. 2017), demographics, maternal characteristics, and child health status.

### 2.5 Control Group

After a random draw of the primary health care centre, the eligible children seen at the health care centre selected as the control group received standard childcare services under Mozambique's National Child Health Protocol (Jhpiego et al. 2011). Children identified with anaemia during follow‐up were referred for medical evaluation and managed according to the national Ministry of Health protocol, which recommends liquid ferrous sulphate (syrup) at a therapeutic dose of 3 mg/kg/day of elemental iron, combined with folic acid supplementation in accordance with age‐specific national guidelines (Ministério da Saúde de Moçambique 2018).

### 2.6 Intervention Group

First, the health professionals were trained on the use of MNPs. For the children seen at the health centre randomly selected for the group intervention, health professionals provided the child;s parents and/or caregivers with 90 MNP sachets to be administered by introducing the contents into solid or semi‐solid foods for 6 months. At the same time, parents or caregivers were instructed by the health centre team on healthy practices for breastfeeding, complementary feeding, and the appropriate use of the sachets. In addition to verbal instructions, caregivers were provided with an illustrated leaflet containing clear visual guidance on the proper preparation and administration of the supplement, aiming to facilitate understanding and promote correct use at home. The WHO recommends supplementation with MNPs from 6 months of life of the child with 90 sachets provided every 6 months for up to 24 months (República de Moçambique Ministério de Saúde 2015). The children in the intervention group were assessed after 6 months to compare against the control group. The period of 6 months for the post‐intervention assessment after 90 sachets is deemed sufficient to attain normal haemoglobin levels(Allen et al. 2009). The sachet formula was in accordance with the composition adopted by UNICEF and currently recommended for intervention studies (Chart S1).

### 2.7 Follow‐up and Assessment of Intervention Adherence After 3 Months

The children were evaluated 3 months after the start of the intervention by the research team, when parents/caregivers returned to the health centre to collect an additional 45 sachets, for a total of 90 sachets dispensed for use over a 6‐month period. Intervention adherence was assessed using a structured questionnaire, which included questions on perceived changes in flavour, colour, or consistency of the food, as well as ease of use, child acceptance, and any adverse effects (e.g., diarrhoea, constipation, general discomfort, or darkening of stools or teeth). The questionnaire also included questions on how caregivers administered the MNPs (e.g., mixing with foods such as porridge); however, these data were collected in a qualitative or self‐reported manner and were not quantified in detail for all participants. The benefits perceived by the mothers were also assessed, including whether they liked giving the supplement and whether they perceived improvements in their child's health.

### 2.8 Assessment of Intervention After 6 Months

After 6 months from the start of the intervention, children aged 12–14 months from both study groups were assessed to determine the effect of supplementation with MNPs. To compare the two groups and perform intra‐group comparisons of results at 6 months against baseline data, the same questionnaire on socioeconomic, demographic, maternal and family aspects, morbidities, and child feeding was applied. Anthropometric data, blood samples by finger prick, and assessment of child food intake were obtained using the same protocol employed at baseline.

### 2.9 Data Analysis

Statistical analyses were performed using the statistical package STATA, version 18.0. Relative and absolute frequencies, means (standard deviation) and medians (interquartile ranges) were calculated for the quantitative variables analysed, with a significance level of *α* = 0.05. The Shapiro–Wilk test was used to verify the distribution of the variables in terms of the observed deviation from that expected under a normal distribution. Student´s *t‐*test and the chi‐square test were employed. A hierarchical model was used for the selection and adjustment of covariates, which were organized into distal, intermediate, and proximal levels according to a previously defined conceptual framework (Figure S1). The intention‐to‐treat analysis compared the mean intra‐group differences on a linear regression model adjusted for child age, wealth index, number of prenatal consultations, maternal age and maternal education. A 95% confidence interval was adopted for all statistical analyses.

### 2.10 Ethics Aspects and Trial Registration

Pursuant to the regulations in Resolution no. 466 of 12/12/2012 of the National Board of Health, providing guidelines on studies involving humans, the project was first submitted to the Mozambique National Committee on Bioethics and approved under permit no. 31/CNBS/2023, and subsequently to the Ethics Committee for Research in Humans of the University of São Paulo, and approved under permit no. 7.309.101. Consent for study participation was obtained within the health service setting, where participants agreed to take part on a voluntary basis by signing a written consent form. Confidentiality and privacy of participants' personal information were guaranteed. The study was registered on the Brazilian Registry of Clinical Trials (REBEC) available at: https://ensaiosclinicos.gov.br/rg/RBR-4r37q7z.

## 3 Results

**Figure 1:**
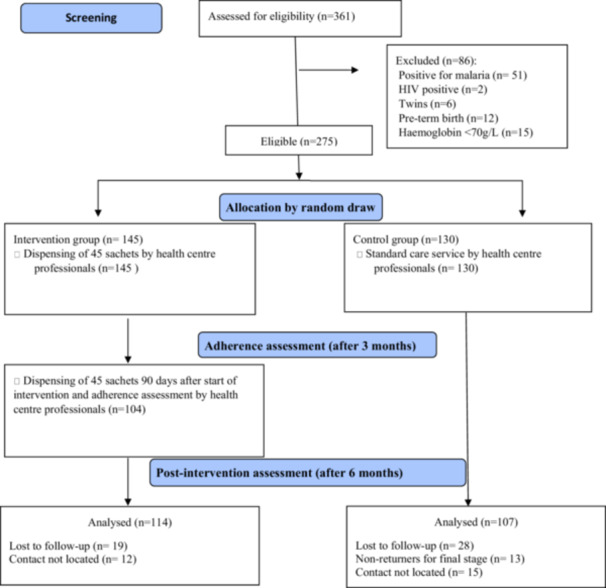
Flow diagram of participant recruitment process (adapted from Schulz 2010).

Of the total 361 participants initially recruited 275 infants eligible for the study were assessed at health centres, designated by random draw as the intervention group (*n* = 145) or control group (*n* = 130), as depicted in the flow diagram (Figure 1).

Baseline socioeconomic, maternal, and environment characteristics of the intervention and control groups are presented in Table 1. There was a statistically significant difference between the groups for the variable's household wealth, maternal age, and maternal education. These variables were included in the adjusted analyses for group comparison.

**Table 1:** Baseline sociodemographic, maternal and environment characteristics by group. Nampula, Mozambique (*n* = 275).

Variable	Frequency *n* (%), *n* = 275	Intervention group *n* = 145	Control group *n* = 130	*p*‐value
No. of residents in household				
≤ 4	108 (39.3)	61 (42.1)	47 (36.2)	0.316
> 4	167 (60.7)	84 (57.9)	83 (63.9)	
Wealth index				**< 0.001**
1st tertile	92 (33.5)	33 (22.8)	59 (45.4)
2nd tertile	92 (33.5)	50 (34.5)	42 (32.3)
3rd tertile	91 (33.5)	62 (42.8)	29 (22.3)
Maternal age ‐ mean (SD)	24.9 (5.6)	25.8 (5.6)	23.9 (5.5)	**0.007**
≤ 19 years	48 (17.5)	15 (10.3)	33 (25.4)	**< 0.001**
> 19 years	227 (82.5)	130 (89.7)	97 (74.6)
Maternal education				
≤ 9 years	148 (53.8)	63 (53.8)	85 (65.4)	**< 0.001**
> 9 years	127 (46.2)	82 (53.8)	45 (34.6)	
Marital status				
Married/Cohabiting	262 (95.3)	136 (93.8)	126 (96.9)	0.222
Single	13 (4.7)	9 (6.2)	4 (3.08)	
Occupation				
Homemaker	255 (92.7)	131 (90.3)	124 (95.4)	0.108
Works outside home	20 (7.3)	14 (9.7)	6 (4.6)	
Number of prenatal consultations				
< 6 consultations	270 (100.0)	143 (53.0)	127 (47.0)	0.565
Type of delivery				
Vaginal	252 (91.9)	133 (91.7)	119 (92.3)	0.873
Caesarean‐section	22 (8.03)	12 (8.3)	10 (7.7)	
Number of children				
≤ 2	162 (58.9)	83 (57.2)	79 (60.8)	
3–4	78 (28.4)	43 (29.7)	35 (26.9)	0.836
≥ 5	35 (12.7)	19 (13.1)	16 (12.7)	
Children under 5				
≤ 1	175 (63.6)	93 (64.1)	82 (63.6)	0.855
> 1	100 (36.4)	52 (35.9)	48 (36.9)	
Head of household			
Father	257 (93.5)	139 (95.9)	118 (90.8)
Mother	18 (6.6)	6 (4.1)	12 (9.2)	0.088
Water treatment			
Treated	105 (38.2)	70 (48.3)	35 (26.9)	**< 0.001**
Untreated	170 (61.8)	75 (51.7)	95 (73.1)
Source of drinking water			
Mains	188 (68.4)	127 (87.6)	61 (46.9)	**< 0.001**
Well/river/rainwater	87 (31.4)	18 (12.4)	69 (53.1)
Conventional cesspit			
Yes	176 (64.0)	99 (68.3)	77 (59.2)	0.119
No	99 (36.0)	46 (31.7)	53 (40.8)

*Note:* Data expressed as mean (standard deviation), or relative frequency (%). *P‐*values obtained using Pearson's chi‐square test or Fisher's Exact test for categorical variables. Values ​​in bold indicate statistical significance.

The comparison between the intervention and control groups at pre‐ and post‐intervention with MNPs is presented in Table 2. After the intervention, the intervention group was found to have a higher mean age than the control group. The mean weight‐for‐age Z‐score was lower in the intervention group than in the control group at endline. Although no differential loss to follow‐up by baseline nutritional status was observed, this finding should be interpreted with caution, given the overall attrition and study design.

**Table 2:** Comparison of neonatal characteristics and child comorbidities for intervention and control groups at baseline and post‐intervention.

	Baseline			Post‐intervention		
Variables	Intervention group *n* = 145	Control group *n* = 130	*p*‐value	Intervention group *n* = 114	Control group *n* = 107	*p‐*value
Mean age (SD)	6. 9 (6.7)	6. 8 (6.7)	0.765	12.6 (0.9)	11.9 (0.8)	**< 0.001** ^**t**^
Sex – *n* (%):						
Male	68 (46.9)	61 (46.9)	0.306	65 (57.0)	53 (49.5)	0.265
Female	77 (53.1)	69 (53.1)		49 (43.0)	54 (50.5)	
Mean gestational age weeks (SD) [95% CI]	38.2 (1.2) [38.02; 38.44]	38.5 (1.2) [38.26; 38.65]	0.127	38.2 (1.3) [37.94; 38.44]	38.6 (1.1) [38.34; 38.77]	**0.033** ^**t**^
Mean birth weight in grams (SD) [95% CI]	3055.4 (484.4) [2975.94; 3134.96]	3013.6 (409.3) [2975.94; 3134.96]	0.443	3074.9 (526.6) [2977.19; 3172.63]	3020.6 (404.5) [2943.04; 3098.08]	0.393^t^
Breastfed in 1st hour – *n* (%) [95% CI]	133 (91.7) [86.09; 95.20]	122 (94.6) [88.33; 96.85]	0.354	105 (92.1) [85.67; 95.79]	102 (95.3) [89.53; 97.99]	0.326
Mean Z‐score weight‐for‐age (SD) [95% CI]	0.05 (1.5) [−0.20; 0.29]	−0.27 (1.4) [−0.53; −0.01]	0.078^t^	0.02 (0.9) [−0.16; 0.20]	0.52 (1.16) [0.29; 0.74]	**< 0.001** ^**t**^
Mean haemoglobin (SD) [95% CI]	102.2 (11.1) [100.4; 104.0]	103.8 (10.7) [102.0; 105.7]	0.213^t^	106.2 (10.6) [104.2; 108.1]	99.5 (11.7) [97.3; 101.8]	**< 0.001** ^**t**^
Anaemia (Hb < 110 g/L) – *n* (%) [95% CI]	107 (73.8) [66.1; 80.3]	86 (66.2) [57.7; 73.7]	0.167	64 (56.1) [47.0; 64.9]	84 (78.5) [69.8; 85.2]	**< 0.001**
Anaemia (Hb < 105 g/L) – *n* (%) [95% CI]	80 (55.2) [47.1; 63.0]	62 (47.7) [39.3; 56.2]	0.215	41 (36.0) [27.8; 45.1]	72 (67.3) [57.9; 75.4]	**< 0.001**
Diarrhoea in past 15 days – *n* (%) [95% CI]	38 (26.2) [19.7; 33.9]	44 (33.9) [26.3; 42.3]	0.969	74 (66.1) [55.8; 73.1]	64 (58.5) [50.3; 68.6]	0.248
Breathing difficulties in past 15 days – *n* (%) [95% CI]	8 (5.6) [2.8; 10.5]	8 (6.2) [3.2; 11.7]	0.833	11 (9.8) [5.5; 16.5]	9 (8.6) [4.5; 15.2]	0.767
Cough in past 15 days – n (%) [95% CI]	77 (53.5) [45.0; 61.0]	71 (54.6) [46.1; 62.9]	0.850	73 (65.8) [54.9; 72.3]	65 (61.9) [51.3; 69.5]	0.555

*Note:* Data expressed as mean (standard deviation) or relative frequency (%) with 95% confidence intervals [95% CI] *P*‐values obtained from Student's *t‐*test or Mann–Whitney test for continuous variables with or without normal distribution, respectively, and Pearson's chi‐square or Fisher's Exact test for categorical variables. ^t^ *p*‐values obtained using Student's *t*‐test. Values ​​in bold indicate statistical significance.

**Figure 2:**
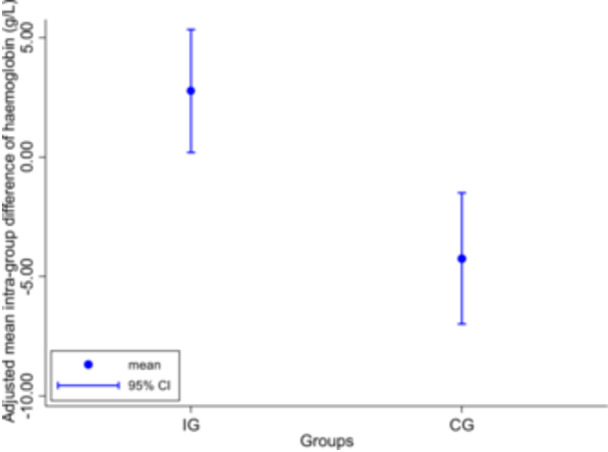
Adjusted mean intra‐group difference of haemoglobin (g/L). Intervention Group (IG) and Control Group (CG). Linear regression adjusted for child age, wealth index, number of pre‐natal consultations, maternal age, and maternal education.

Post‐intervention, the prevalence of anaemia was lower in the intervention group than in the control group. After adjusting for age, wealth index, number of prenatal consultations, maternal age, and maternal education, mean haemoglobin concentration increased by 2.8 g/L (95% CI 0.18; 5.36) in the intervention group, whereas it decreased by 4.3 g/L (95% CI −0.78; −4.97) in the control group (Figure 2), suggesting a positive effect of MNP use on intra‐group Hb levels.

The results of the assessment of adherence to home supplementation with MNPs 90 days after the start of the intervention are presented in Table 3. More than half of the children consumed all of the sachets and 70% used over 75% of the sachets prescribed. Regarding frequency of use, 40% of the children received MNPs daily. The main reasons for partial consumption or non‐daily administration of sachets were rejection by the child and because the mother forgot. No adverse effects were reported. The vast majority of mothers introduced the MNPs into porridge, a practice not recommended by health professionals. The level of acceptability reported by mothers was predominantly positive, with 39% of participants rating this as excellent. In 94% of cases, the mother was the person who administered the MNPs to the child (Table 3).

**Table 3:** Adherence to home supplementation with MNPs after 90 days of intervention. Nampula, Mozambique, (*n* = 104).

Variable	Frequency *n* (%)
MNP use:	
All sachets	55 (53.4)
< 75% used	31 (29.8)
≥ 75% used	73 (70.2)
Mean (SD) sachets unused	3.8 (6.0)
75% of sachets unused	6 (13.3)
Frequency of MNP administration:	
Daily	41 (39.8)
1 time a week	32 (31.1)
4 times a week	30 (29.1)
Reason for partial use:	
Child rejected	25 (24.0)
Mother forgot	20 (19.2)
Adverse effects	3 (2.9)
Used > 1 per day:	
No	99 (95.2)
Yes	5 (4.8)
MNP in savoury food:	
No	34 (32.7)
Yes	70 (67.3)
MNP in fruit smoothie:	
No	102 (98.1)
Yes	2 (1.9)
MNP in porridge:	
No	7 (6.7)
Yes	97 (93.3)
MNP in juice:	
No	104 (100.0)
MNP in water:	
No	104 (100.0)
Food to which MNP most commonly added:	
Savory food/porridge	50 (48.1)
Porridge	54 (51.9)
Level of acceptability reported by mother:	
Excellent	40 (38.5)
Good	32 (30.8)
Fair	30 (28.9)
Poor	2 (1.9)
Administered by other person:	
No	98 (94.2)
Yes	6 (5.8)

*Note:* Data expressed as median (interquartile range) or relative frequency (%).

On the intervention efficacy analysis, only participants who administered 75% of the MNPs prescribed were included (Tables S1 and S2). Mean difference in Hb concentration on the efficacy analysis (3.2 g/L [95% CI: 0.51 to 5.88]) in the IG versus a decrease of 4.4 g/L (95% CI −0.84;−5.01) in the CG was slightly higher than the difference on the intention‐to‐treat analysis. These results show that greater adherence to daily use of MNPs was associated with a greater impact on Hg concentrations (Figure S2).

## 4 Discussion

This first pragmatic clinical trial conducted in Mozambique demonstrated that the primary outcome, an increase in mean blood haemoglobin concentration, was achieved following the implementation of home fortification with MNPs, with a concurrent reduction in anaemia prevalence among children in the first year of life. The prevalence of anaemia (defined both by the traditional WHO cutoff of 110 g/L and the new threshold of 105 g/L) (World Health Organization 2011; World Health Organization 2024) was lower in the intervention group.

In this study, after adjustment for child and maternal characteristics, the mean change in haemoglobin from baseline to follow‐up was higher in the intervention group than in the control group, indicating a positive effect of MNPs. These findings are consistent with evidence observed in other settings. In a study performed in Pakistan, MNP supplementation promoted a significant mean increase in haemoglobin of 3.2 g/L (Soofi et al. 2013). Similarly, in Ghana, Adu‐Afarwuah et al. reported improved Hb levels and lower anaemia rates after long‐term use of MNPs (Adu‐Afarwuah et al. 2007). A randomized clinical trial (RCT) involving over 4000 children in India found that home fortification of complementary foods with micronutrient powders led to modest increases in Hb and reductions in the prevalence of anaemia and diarrhoea among children aged 6–18 months (Young et al. 2021). A national study of home fortification of complementary food in Brazil in 24 large primary healthcare centres observed a positive impact of the use of MNPs on haemoglobin levels in children under 1 year of age (Cardoso et al. 2016).

A systematic review and meta‐analysis including 30 RCTs showed that home fortification with MNPs represents an effective method for improving haemoglobin, iron and zinc levels (Nikooyeh and Neyestani 2021). Another systematic review and meta‐analysis of studies on micronutrient fortification and supplementation interventions on health and development outcomes among children under 5 in low‐ and middle‐income countries found that supplementation with MNPs was associated with a lower risk of anaemia compared to no intervention or placebo (Tam et al. 2020).

With regard to adherence to MNPs in the present study, children who consumed at least 75% of the prescribed sachets showed a more pronounced effect of the intervention on Hb concentrations, confirming the effect of MNPs in increasing Hb levels. Despite this positive outcome, most mothers mixed the MNPs into porridge, which is an accessible and widely consumed food preparation in Nampula. However, administering MNPs in the form of porridge is not recommended because of resulting changes in flavour and poor acceptability by children, as well as its low nutritional density (Ganhão et al. 2017). In general, complementary food in Cabo Delgado province, Mozambique, fails to meet WHO recommendations: only 23% of children aged 6–23 months receive a minimally acceptable diet, with low food diversity and irregular meals (Marroda et al. 2023). A strategy for improving dietary patterns is to combine MNP dispensing with educational actions on complementary food, emphasizing the importance of a varied diet and the use of accessible local ingredients (Sokhela et al. 2023).

In a systematic review on adherence to and acceptability of home fortification with MNPs in children aged 6–23 months, adherence ranged from 50% to 96% in the studies reviewed (De Barros and Cardoso 2016). Overall, adherence was considered satisfactory in most studies, and the authors noted that factors such as proper guidance for caregivers, absence of perceived adverse effects, and ease of use of the sachets were determinants for intervention success (De Barros and Cardoso 2016). These results highlight the importance of actions to raise awareness and provide guidance for caregivers, who likely played a fundamental role in attaining the levels of adherence seen in Nampula.

In addition, studies such as those by Young et al. (2021) in India and Adu‐Afarwuah et al. (2008) in Ghana report adherence rates of between 65% and 75%, closely resembling those observed in Nampula, suggesting that well‐structured interventions can achieve good acceptance, even in situations of socioeconomic vulnerability (Young et al. 2021; Adu‐Afarwuah et al. 2008). By contrast, Soofi et al. (2013) in Pakistan found adherence of under 60%, attributed to caregiver concerns over possible adverse effects (Soofi et al. 2013).

The present study has some limitations, including finite human resources and materials, and the use of haemoglobin concentration as the sole biochemical indicator. Biomarkers of the body's iron reserves, such as blood levels of ferritin, C‐reactive protein, and transferrin receptor, allow assessment of the impact of MNP use on iron status, the leading cause of anaemia in healthy neonates. In addition, the allocation was conducted at the level of health facilities, and only two centres were included (one intervention and one control), resulting in a very limited number of clusters. The fact that the study was conducted in only two centres, with a single cluster per study arm, may affect not only the external validity (generalizability) but also the internal validity of the findings. In this design, the intervention is fully confounded with the study site, making it impossible to disentangle the effects of the intervention from inherent differences between health centres. Differences in centre‐specific practices, population characteristics, and the presence of unmeasured confounders may therefore have influenced the observed results. Moreover, because allocation occurred at the cluster level, but analyses were conducted at the individual level, residual confounding and clustering effects could not be adequately accounted for. Additionally, the restricted number of sites may introduce selection bias and limit the representativeness of the sample. Therefore, caution is warranted when interpreting these findings, which should be considered as preliminary and hypothesis‐generating, and future multicentre studies with a larger number of clusters are needed to confirm the robustness and broader applicability of the results.

Another limitation relates to the criteria used to define the adjustment set in the analytical models. Although covariate selection was partially informed by statistically significant baseline differences between groups, we also applied a predefined hierarchical conceptual framework to guide variable inclusion according to distal, intermediate, and proximal associated factors. Nevertheless, reliance in part on statistical significance may not fully capture the underlying causal structure. Therefore, residual confounding cannot be entirely ruled out. Despite these limitations, the study has several strengths, such as being the first pragmatic trial to assess the effect of MNPs in primary healthcare in Mozambique, particularly in Nampula, a province with one of the highest rates of food insecurity and childhood anaemia in the country. The use of the updated cutoff for classifying anaemia by the WHO, both the traditional cutoff (Hb < 110 g/L) and the newly proposed threshold (Hb < 105 g/L), with the sample power obtained, suggests that the dispensing of MNPs under the standard child healthcare service in Mozambique is effective for increasing Hb levels in children under 1 year, in an African context. In addition, the results of the analysis restricted to participants with higher adherence underscored the importance of consistent and correct use of MNPs, promoting an even more marked increase in haemoglobin levels in the intervention group. The high level of acceptability of the use of MNPs in complementary food demonstrates the practical feasibility of implementing the strategy in similar settings, although challenges such as food rejection, forgetfulness, and inappropriate use indicate the need for clearer guidance to families.

In conclusion, the sustainable and systematic integration of MNPs into primary healthcare in Mozambique, alongside WHO‐recommended child survival interventions such as breastfeeding support, vaccination, morbidity prevention and control, growth monitoring, and the promotion of healthy complementary feeding practices, combined with ongoing training for health professionals, may represent a promising strategy to control anaemia in infants and improve nutritional status and child development.

## Author Contributions

A.R.M.G. and M.A.C. designed the research study. A.R.M.G. performed the data collection and processing. A.R.M.G. and M.A.C. performed the data analysis and interpretation of the results. A.R.M.G. wrote the initial version of the manuscript with input from M.A.C. All authors contributed to revisions of the manuscript and approved the final version.

## Conflicts of Interest

The authors declare no conflicts of interest.

## Supporting information

Supporting File

## Data Availability

The data that support the findings of this study are available on request from the corresponding author. The data are not publicly available due to privacy or ethical restrictions.
