# Supplementary material for: Effect of Home‐Based Multiple Micronutrient Powder Fortification on Haemoglobin Levels in Infants in Nampula, Mozambique: A Pragmatic Clinical Trial
Source: Matern Child Nutr. 2026 Jun 19;22(3):e70210. doi: 10.1111/mcn.70210 (PMC13280800; doi:10.1111/mcn.70210)
Supplement: Supplementary file 1 — Supporting File [file MCN-22-e70210-s001.docx]

**Supplementary Material 1**

**Chart S1. Nutrient composition per 1 g sachet of multiple micronutrient powder (MNP)**

| Micronutrient | Quantity |
| --- | --- |
| Iron | 10 mg |
| Zinc | 4.1 mg |
| Folic acid | 150 µg |
| Vitamin A | 400 µg RE |
| Vitamin C | 30 mg |
| Vitamin D3 | 5 µg |
| Vitamin E | 5 mg TE |
| Vitamin B1 | 0.5 mg |
| Vitamin B2 | 0.5 mg |
| Vitamin B6 | 0,5 mg |
| Vitamin B12 | 0.9 µg |
| Niacin | 6 mg |
| Copper | 0.56 mg |
| Iodine | 90 µg |
| Selenium | 17 µg |

Source: Mix Me Sachet, produced by DSM Nutritional Products Europe Ltd.

**Table S1. Sociodemographic, maternal and environmental characteristics of participants according to groups at baseline for effectiveness analysis. Nampula, Mozambique (n = 266).**

| Variable | | Frequency n (%), median (IQR) and mean (SD) total n=266 | Intervention group  n=136 | Control group  n=130 | p-value |
| --- | --- | --- | --- | --- | --- |
| Number of residents in household | |  |  |  |  |
| Up to 4 people | | 103 (38.7) | 56 (41.2) | 47 (36.2) | 0.401 |
| More than 4 people | | 163 (61.3) | 80 (58.8) | 83 (63.9) |  |
| Wealth index |  |  |  | **<0.001** |  |
| 1st tercile | 90 (33.8) | 31 (22.8) | 59 (45.4) |  |  |
| 2nd tercile | 88 (33.1) | 46 (33.8) | 42 (32.3) |  |  |
| 3rd tercile | 88 (33.1) | 59 (43.4) | 29 (22.3) |  |  |
| Maternal age | 24.9 (5.6) | 25.8 (5.5) | 23.9 (5.5) | **0.007** |  |
| mean (SD) | 46 (17.3) | 13 (9.6) | 33 (25.4) | **<0.001** |  |
| ≤ 19 years | 220 (82.7) | 123 (90.4) | 97 (74.6) |  |  |
| > 19 years | | 8.4 (3.6) | 9. 3 (3.3) | 7. 6 (3.7) | **<0.001** |
| Mother's schooling | | 145 (53.8) | 60 (44.1) | 85 (65.4) | **<0.001** |
| mean (SD) | | 127(46.2) | 76 (55.9) | 45 (34.6) |  |
| ≤ 9 years | |  |  |  |  |
| > 9 years | | 254 (95.5) | 136 (93.8) | 126 (96.9) | 0.270 |
| Marital status | | 12 (4.5) | 9 (6.2) | 4 (3.1) |  |
| Married/lives together | |  |  |  |  |
| Single | | 246 (92.5) | 122 (89.7) | 124 (95.4) | 0.079 |
| Mother's occupation | | 20 (7.5) | 14 (10.3) | 6 (4.6) |  |
| Housewife | |  |  |  |  |
| Works outside | | 261 (100.0) | 134 (51.3) | 127 (48.7) | 0.615 |
| Number of antenatal visits | |  |  |  |  |
| < 6 consultations | | 243 (91.7) | 124 (91.2) | 119 (92.3) | 0.752 |
| Type of birth (n=265) | | 22 (8.3) | 12 (8.8) | 10 (7.7) |  |
| Normal | |  |  |  |  |
| Caesarean section | | 156 (58.7) | 77 (56.6) | 79 (60.8) |  |
| Number of children | | 76 (28.5) | 41 (30.2) | 35 (26.9) | 0.786 |
| ≤ 2 | | 34 (12.8) | 18 (13.2) | 16 (12.3) |  |
| 3-4 | |  |  |  |  |
| ≥ 5 | | 167 (62.8) | 85 (62.5) | 82 (63.1) | 0.193 |
| Children under 5 | | 99 (37.2) | 51 (37.5) | 48 (36.9) |  |
| ≤ 1 |  |  |  |  |  |
| >1 | 249 (93.6) | 131 (96.3) | 118 (90.8) |  |  |
| Head of household | 17 (6.4) | 5 (3.7) | 12 (9.2) | 0.064 |  |
| Father |  |  |  |  |  |
| Mother | 98 (36.8) | 63 (46.3) | 35 (26.9) | **<0.001** |  |
| Water treatment | 168 (63.2) | 73 (53.7) | 95 (73.1) |  |  |
| Treated |  |  |  |  |  |
| Untreated | 182 (68.4) | 121 (89.0) | 61 (46.9) | **<0.001** |  |
| Source of drinking water | 84 (31.6) | 15 (11.0) | 69 (53.1) |  |  |
| Public |  |  |  |  |  |
| Well/river/rain | 171 (64.3) | 94 (69.1) | 77 (59.2) | 0.093 |  |
| Conventional cesspit | 95 (35.7) | 42 (30.9) | 53 (40.8) |  |  |

Data presented as mean (standard deviation) or relative frequency (%). P-values obtained from Pearson's chi-squared test for categorical variables.

**Table S2. Per protocol analysis: comparison of neonatal characteristics and children's comorbidities between the intervention and control groups. before and after the intervention. Nampula. Mozambique.**

| **Variables** | **Baseline** | | | | **Post-intervention** | | | |
| --- | --- | --- | --- | --- | --- | --- | --- | --- |
|  | **Frequency n (%) and mean (SD) total n=266** | **Intervention group**  **n=136** | **Control**  **group**  **n=130** | **p-value** | **Frequency n (%) and mean (SD) total n=212** | **Intervention group**  **n=105** | **Control group**  **n=107** | **p-value** |
| Mean age (SD) | 6. 9 (6.8) | 6. 9 (6.7) | 6. 8 (6.7) | 0.888t | 12.2 (0.9) | 12.6 (0.9) | 11.9 (0.8) | **<0.001** |
| Sex– n (%): |  |  |  |  |  |  |  |  |
| Male | 134 (50.4) | 65 (47.8) | 61(46.9) | 0.386 | 111 (52.4) | 59 (56.2) | 52 (48.6) | 0.268 |
| Female | 132 (49.6) | 71 (52.2) | 69 (53.1) |  | 101 (47.6) | 49 (43.8) | 55 (51.4) |  |
| Gestational age (weeks) |  |  |  |  |  |  |  |  |
| Mean (SD)[95% CI] | 38.3 (1.2)  [38.2; 38.5] | 38.2 (1.3)  [37.9; 38.4] | 38.5 (1.1)  [38.3; 38.7] | 0.084t | 38.3 (1.3)  [38.2; 38.5] | 38.1 (1.4)  [38.9; 38.4] | 38.6 (1.1)  [38.3; 38.8] | **0.019^t^** |
|  |  |  |  |  |  |  |  |  |
| Birth weight (grams)[95% CI] | 3026.7 (449.4)  [2972.4; 3080.9] | 3039.3 (485.9)  [2956.9; 3121.7] | 3013.6 (409.3)  [2942.6; 3084.6] | 0.643^t^ | 3037.9 (471.5)  [2974.1; 3101.8] | 3055.6 (532.6)  [2952.5; 3158.7] | 3020.6 (404.5)  [2943.0; 3098.1] | 0.589^t^ |
|  |  |  |  |  |  |  |  |  |
| Breastfed within the first hour [95% CI] |  |  |  |  |  |  |  |  |
| Yes | 247 (93.2) | 125 (91.9) | 122 (94.6) | 0.389 | 199 (93.9) | 97 (92.4) | 102 (95.3) | 0.326 |
| No | 18 (6.8) | 11 (8.1) | 5 (5.4) |  | 13 (6.1) | 8 (7.6) | 5 (4.7) |  |
|  |  |  |  |  |  |  |  |  |
| Mean weight-for-age z-score (SD) [95% CI] | -0.1 (1.5)  [-0.3;0.1] | -0.2 (1.5)  [-0.3;0.3] | -0.27 (1.4)  [- 0.5; 0.1] | 0.115^t^ | 0.3 (1.1)  [0.1; 0.7] | 0.1 (0.9)  [-0.1; 0.2] | 0.5 (1.2)  [0.3; 0.7] | **<0.002^t^** |
| Mean haemoglobin (g/L) (SD) [95% CI] | 102.9 (11.0)  [101.6; 104.3] | 102.1 (11.3)  [100.2; 104.0] | 103.8 (10.6)  [101.9; 105.7] | 0.208^t^ | 103.0 (11.6)  [101.4; 104.6] | 106.7 (10.3)  [104.8; 108.8] | 99.4 (11.7)  [97.1; 101.6] | **<0.001^t^** |
|  |  |  |  |  |  |  |  |  |
| Anaemia (Hb <110g/L) [95% CI] | 185 (69.5)  [63.8; 74.8] | 99 (72.8)  [64.8; 79.6] | 86 (66.2)  [57.7; 73.7] | 0.239 | 142 (67.0)  [60.4; 73.0] | 58 (55.2)  [45.7; 64.4] | 84 (78.5)  [69.8; 85.2] | **<0.001** |
|  |  |  |  |  |  |  |  |  |
| Anaemia (Hb <105g/L) [95% CI] | 135 (50.8)  [44.8; 56.7] | 73 (53.7)  [45.3; 61.8] | 62 (47.7)  [39.3; 56.2] | 0.329 | 108 (50.9)  [44.3; 57.6] | 36 (34.3)  [25.9; 43.8] | 72 (67.3)  [57.9; 75.4] | **<0.001** |
| Diarrhoea in the last |  |  |  |  |  |  |  |  |
| 15 days (n=264) [95% CI] | 76 (28.8)  [23.5; 34.3] | 39 (26.1)  [21.7; 36.8] | 37 (28.5)  [21.4; 36.8] | 0.908 | 129 (61.4)  [54.1; 67.2] | 68 (65.4)  [57.7; 73.7] | 61 (57.5)  [47.6; 66.0] | 0.243 |
|  |  |  |  |  |  |  |  |  |
| Breathing difficulties in the last 15 days (n=265) [95% CI] | 16 (6.0)  [3.7; 9.5] | 8 (5.9)  [3.0; 11.2] | 8 (6.2)  [3.2; 11.7] | 0.833 | 18 (8.7)  [5.4; 13.0] | 9 (8.6)  [4.6; 15.5] | 8 (9.6)  [3.8; 14.1] | 1.000 |
|  |  |  |  |  |  |  |  |  |
| Cough in the last 15 days (n=265) [95% CI] | 123 (46.4)  [40.4; 52.2] | 64 (47.4)  [38.9; 55.8] | 59 (45.4)  [37.1; 53.9] | 0.741 | 135 (65.2)  [57.0; 69.9] | 70 (68.6)  [57.2; 74.9] | 65 (61.9)  [51.3; 69.5] | 0.310 |

Data presented as mean (standard deviation) or relative frequency (%). P-values obtained from the t-test or Mann-Witney test for continuous variables with and without normal distribution, respectively, and Pearson's chi-squared test for categorical variables. ^t^ t-test.

**Prenatal and obstetric care**

Prenatal consultations, type of delivery, parity

**Environmental characteristics**

Drinking water source, water treatment, sanitation facilities, number of households

**Child Nutritional Status and Comorbidities**

Diarrhoea, pneumonia, fever, cough, breathing difficulties, underweight, overweight

**Characteristics at birth and breastfeeding**

Gestational age, sex of the child, weight and length at birth, initiation of breastfeeding, introduction of complementary foods, use of supplements

**Anaemia**

**Demographic and socioeconomic characteristics**

Residents in the household, family wealth index, maternal age, mother's and father's education, marital status, head of household, occupation, receipt of financial assistance

**Distal level**

**Intermediate level**

**Proximal level**

**Figure S1. Conceptual framework for selection of covariates in relation to anaemia risk in children based on previous studies (Cardoso MA et al, 2024).**

**Figure S2- Adjusted mean intra-group difference in haemoglobin (g/L), considering only participants who adhered to 75% of the MNPs. Intervention group (IG) and control group (CG). Linear regression adjusted for child's age, wealth index, number of antenatal visits, maternal age and maternal schooling.**
